# Long-term exercise training down-regulates m^6^A RNA demethylase FTO expression in the hippocampus and hypothalamus: an effective intervention for epigenetic modification

**DOI:** 10.1186/s12868-022-00742-8

**Published:** 2022-09-26

**Authors:** Shu-Jing Liu, Tong-Hui Cai, Chun-Lu Fang, Shao-Zhang Lin, Wen-Qi Yang, Yuan Wei, Fu Zhou, Ling Liu, Yuan Luo, Zi-Yi Guo, Ge Zhao, Ya-Ping Li, Liang-Ming Li

**Affiliations:** 1 Center for Scientific Research and Institute of Exercise and Health, Guangzhou Sport University, Guangzhou, China; 2 The Third Affiliated Hospital of Guangzhou Medical University, Guangzhou, 510260 China

**Keywords:** Exercise, Hippocampus, Hypothalamus, RNA-sequencing, FTO

## Abstract

**Background:**

Exercise boosts the health of some brain parts, such as the hippocampus and hypothalamus. Several studies show that long-term exercise improves spatial learning and memory, enhances hypothalamic leptin sensitivity, and regulates energy balance. However, the effect of exercise on the hippocampus and hypothalamus is not fully understood. The study aimed to find epigenetic modifications or changes in gene expression of the hippocampus and hypothalamus due to exercise.

**Methods:**

Male C57BL/6 mice were randomly divided into sedentary and exercise groups. All mice in the exercise group were subjected to treadmill exercise 5 days per week for 1 h each day. After the 12-week exercise intervention, the hippocampus and hypothalamus tissue were used for RNA-sequencing or molecular biology experiments.

**Results:**

In both groups, numerous differentially expressed genes of the hippocampus (up-regulated: 53, down-regulated: 49) and hypothalamus (up-regulated: 24, down-regulated: 40) were observed. In the exercise group, increased level of N6-methyladenosine (m^6^A) was observed in the hippocampus and hypothalamus (*p* < 0.05). Furthermore, the fat mass and obesity-associated gene (*FTO*) of the hippocampus and hypothalamus were down-regulated in the exercise group (*p* < 0.001). In addition, the *Fto* co-expression genes of the mouse brain were studied and analyzed using database to determine the potential roles of exercise-downregulated FTO in the brain.

**Conclusion:**

The findings demonstrate that long-term exercise might elevates the levels of m^6^A-tagged transcripts in the hippocampus and hypothalamus via down-regulation of FTO. Hence, exercise might be an effective intervention for epigenetic modification.

## Introduction

The brain is the master organ of the central nervous system that modulates body organ functioning. The hippocampus and hypothalamus are parts of the brain crucial for the body's physiological functions. The hippocampus is a highly plastic region associated with stress response, learning, and memory [1, 2], and the hypothalamus is a critical central regulatory center for blood sugar, energy balance, and water balance [3]. Dysfunction of these parts can lead to adverse effects.

Exercise targets various aspects of brain function and broadly influences brain health. Studies on humans and animals suggested that physical exercise improves spatial learning and memory [4, 5]. Some studies reported that exercise controls obesity by enhancing hypothalamic leptin sensitivity [6, 7]. However, the cellular and molecular effects of exercise on the hippocampus and hypothalamus remain unknown. Therefore, it is important to study the effect of exercise on gene expression in the brain, and find a non-drug method to maintain brain health.

Recently, epigenetic regulation in various biological functions and pathogenesis of diseases has gained attention. m^6^A is one of the most common post-transcriptional RNA modifications in mRNA, represents another novel epigenetic marker, play critical roles in the regulation of gene expression. Through mutual interplay with methyltransferases, demethylases and m^6^A binding proteins to balance the m^6^A level, and to insure the mRNA transcripts can be properly spliced, transported, transcripts, and degraded [8]. Exercise as a lifestyle intervention can fine-tune gene expressions and biological processes via epigenetic modifications [9]. In the present study, a transcriptome profiling technology RNA-sequencing was used to identify differentially expressed genes of the hippocampus and hypothalamus in exercise training models. In the exercise group, an increased level of m^6^A was observed in the hippocampus and hypothalamus. Furthermore, the m^6^A RNA methylation regulator expression was assessed. Bioinformatic analysis showed that the *Fto* gene was down-regulated in the hippocampus and hypothalamus, which responds to exercise. Based on previous bioinformatics analyses, we further confirm *Fto* expression and other m^6^A RNA methylation regulators in the hippocampus and hypothalamus of exercise mice using qPCR and western blot analysis.

*FTO* is associated with an increased risk of diabetes and obesity [10]. Recently, the *FTO* gene and its expression product have attracted widespread interest due to its identification as an m^6^A RNA demethylase [11–13]. FTO is highly expressed in the brain and likely involved in many nuclear RNA processing events, such as mRNA translation, splicing, and metabolism [11, 13]. Previous study showed that highly intensive exercise decreases the skeletal muscle *FTO* mRNA [14]. However, the evidence on the effects of long-term exercise on *FTO* expression is scarce. Hence, evidence on the molecular biological mechanisms of exercise-induced changes of FTO-m^6^A expression on brain function and the biological process has been provides in this study.

## Experimental procedure

### Animals and diet administration

**Fig. 1:**
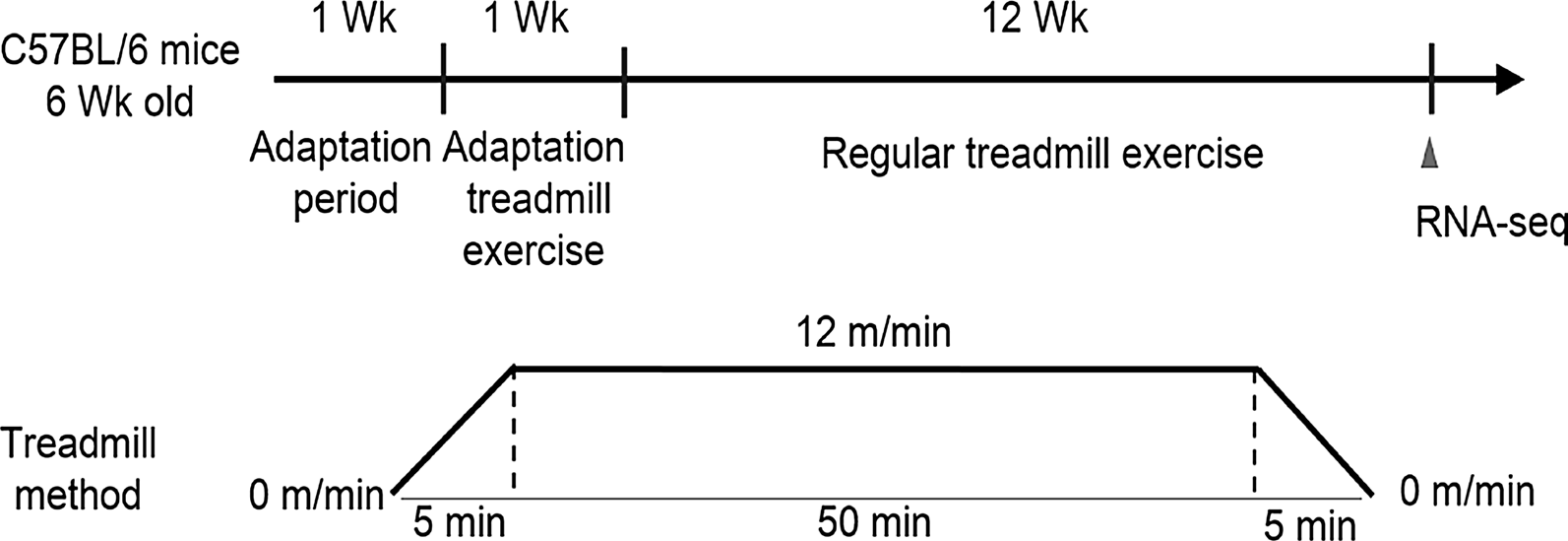
Construction of 12-week exercise training model in exercise mice and exercise protocol

The C57BL/6 mice were provided by the Experimental Animal Center of Sun Yat-sen University (Guangzhou, China). Eight-week-old male C57BL/6 J mice were randomly divided into sedentary (n = 24) and exercise (n = 24) groups. The mice in the exercise group were trained on a treadmill 5 days per week for 12 weeks (Fig. 1). The schedule was as follows: 5 min of warm-up at 0–12 m/min, 50 min of the main exercise at 12 m/min (moderate-intensity exercise with 75% maximum oxygen consumption), and 5 min of cool down at 12–0 m/min [15]. The mice in the sedentary group were controls. And the control mice were exposed to treadmill noise and vibration without runing. The mice were fed a standard diet and water ad libitum in a 12 h-light/12 h-dark cycle at the Guangzhou Sport University. Five days after the final exercise training, mice were assessed for body composition and metabolic status. The mice were euthanized under anesthesia (sodium pentobarbital 50 μg/g) for collection of the hippocampus and hypothalamus tissues. This study was approved by the Institutional Animal Care and Use Committee of Guangzhou Sport University (2021DWLL-05).

### Metabolic assessments

Mice were placed in an Oxymax Comprehensive Lab Animal Monitoring System (Columbus Instruments, USA) to detect the oxygen consumption (VO_2_), the carbon dioxide production (VCO_2_), respiratory exchange ratio (RER), energy intake, and energy expenditure. The body composition was assessed using the EchoMRI quantitative magnetic resonance (QMR) method (EchoMRI-500H, USA).

### Library construction for RNA-sequencing

The quantity and purity of the total RNA were analyzed using the Bioanalyzer 2100 and RNA 1000 Nano LabChip Kit (Agilent, USA) with RIN number > 7.0. Poly(A) RNA was purified from the total RNA (5 μg) using poly-T oligo-attached magnetic beads with two rounds of purification. Subsequently, the mRNA was fragmented into small pieces using divalent cations under elevated temperatures. The cleaved RNA fragments were then reverse transcribed using the mRNA Seq sample preparation kit (Illumina, USA) to create the final cDNA library [the average insert size for the libraries was 300 bp (± 50 bp)]. In addition, the paired-end sequencing was performed on an Illumina Novaseq™ 6000 (LC-Bio Technology CO., Ltd., Hangzhou, China) following the vendor's recommended protocol.

### RNA extraction and quantitative real-time PCR (qPCR)

Total RNA from the mouse hippocampus and hypothalamus tissues were extracted using HiPure Universal RNA Kit (Magen, China). The cDNA was synthesized from 1 μg of total RNA using PrimeScript™ RT Reagent Kit with gDNA Eraser (TaKaRa, Japan). qPCR analysis was conducted using TB Green^®^ Premix Ex Taq™ (TaKaRa, Japan) with Applied Biosystems 7500 Real**-**Time PCR System (Thermo Fisher Scientific, USA). The relative cycle threshold (CT) values were normalized using *β-actin*. All primers used for qPCR are listed in Table 1.

**Table 1:** Mouse specific primer sequences used for qPCR (*β-actin* for the housekeeping as an internal control)

Gene	Forward sequence	Reverse sequence
*Fto*	GCAGAGCAGCCTACAACGTGAC	CCAACATGCCAAGTATCAGGATCTC
*Alkbh5*	GGGTGTCGGAACCTGTGCTTTCTC	GCAATGTGGAGCTGCTCAGGGAT
*Mettl3*	GAGCTAGGATGTCGGACACG	GCACGGGACTATCACTACGG
*Mettl14*	GGGAAGGATTGGACCTTGGG	ACCCCACTTTCGCAAGCATA
*Wtap*	TCTTGTCATGCGGCTAGCAA	GCGTAAACTTCCAGGCACTC
*Rbm15*	GAAGAGCCAGAGCGACAAGC	GAGGTCACCCTGCAACAGAT
*Zc3h13*	GGAAGTCCAAGAAACGCTATAGA	CGAGATTCCTGTGGCCGTAC
*Ythdf1*	ACAGTTACCCCTCGATGAGTG	GGTAGTGAGATACGGGATGGGA
*Ythdf3*	TGACAACAAACCGGTTACCA	TGTTTCTATTTCTCTCCCTACGC
*Ythdc2*	GGTCCGATCAATCATCTGT	GAAGTAACGAATAGGCATGT
*Ythdf2*	GAGCAGAGACCAAAAGGTCAAG	CTGTGGGCTCAAGTAAGGTTC
*Hnrnpc*	GCCAGCAACGTTACCAACAA	TGAACAGAGCAGCCCACAAT
*Ythdc1*	CCAAAGCAAAGGGTGTATGGTC	TCATTCCAGGGATTGGTGAGAT
*β-actin*	TGGTCGTCGACAACGGCTC	CCATGTCGTCCAGTTGGTAAC

### Protein extraction and western blot

The mouse hippocampus and hypothalamus tissues were lysed on ice using RIPA lysis buffer (100 mm NaCl, 20 mm Tris, pH8.0, 1 mm EDTA, pH8.0, 0.5% Triton X-100, and 0.5% Nonidet P-40) containing a protease and phosphatase inhibitor cocktail (Beyotime Biotechnology, China). These tissues were quantified using the BCA Protein Assay Kit (Pierce, Germany). The same amount of protein (15 μg) was resolved on a 12% SDS-PAGE under a denaturing condition, transferred onto a PVDF membrane, and blocked in 5% non-fat milk. The blots were cut prior to hybridisation with antibodies. After the tissues were incubated with FTO antibody (Cat#: 98768, Santa Cruz Biotech, USA) or β-actin antibody (Cat#: 60008-1-Ig, ProteinTech Group, USA) overnight at 4 °C and secondary antibody (Peroxidase-conjugated Affinipure Goat Anti Mouse/Rabbit IgG, ProteinTech Group, USA) for 2 h at room temperature, the bands were exposed using enhanced chemiluminescence (Pierce, USA) and X-ray film. Quantitative data were obtained using ImageJ software.

### m^6^A level

The m^6^A RNA methylation status of the mouse hippocampus and hypothalamus were detected using enzyme-linked immunoassay (ELISA) with an EpiQuik™ m^6^A RNA Methylation Quantification Kit (Epigebtek, China) following the manufacturer's protocol. The detected signal was quantified by reading the absorbance in a microplate spectrophotometer. The amount of m^6^A is proportional to the OD intensity measured.

### Bioinformatic analysis of *Fto* gene co-expression network

The transcriptome and expression profiles of *Fto* and other m^6^A RNA methylation regulators were analyzed using the R package “Limma” (R software version R3.6.3). The *Fto* gene co-expression network in the mouse brain were analyzed using the coexpedia database. To explore the functional annotation and pathway enrichment of the co-expression network in the brain, the Gene Ontology (GO) and Kyoto Encyclopedia of Genes and Genomes (KEGG) databases were analyzed using the Database for Annotation, Visualization and Integrated Discovery (DAVID) v6.8 online analysis tool.

### Statistical analyses

All experimental data were analyzed using the SPSS 20.0 software, and the results were expressed as mean ± SEM. Kolmogorov–Smirnov and Shapiro–Wilk normality tests were performed and homogeneity of variance was tested with the Levene. Statistical significance of differences between the two groups were calculated using Student's t-test. When variables did not fulfill assumptions of normality, the Kruskal–Wallis test was applied. p < 0.05 were considered significant and marked with an asterisk (*).

## Results

### Characterization of mice with 12-week exercise training

**Fig. 2:**
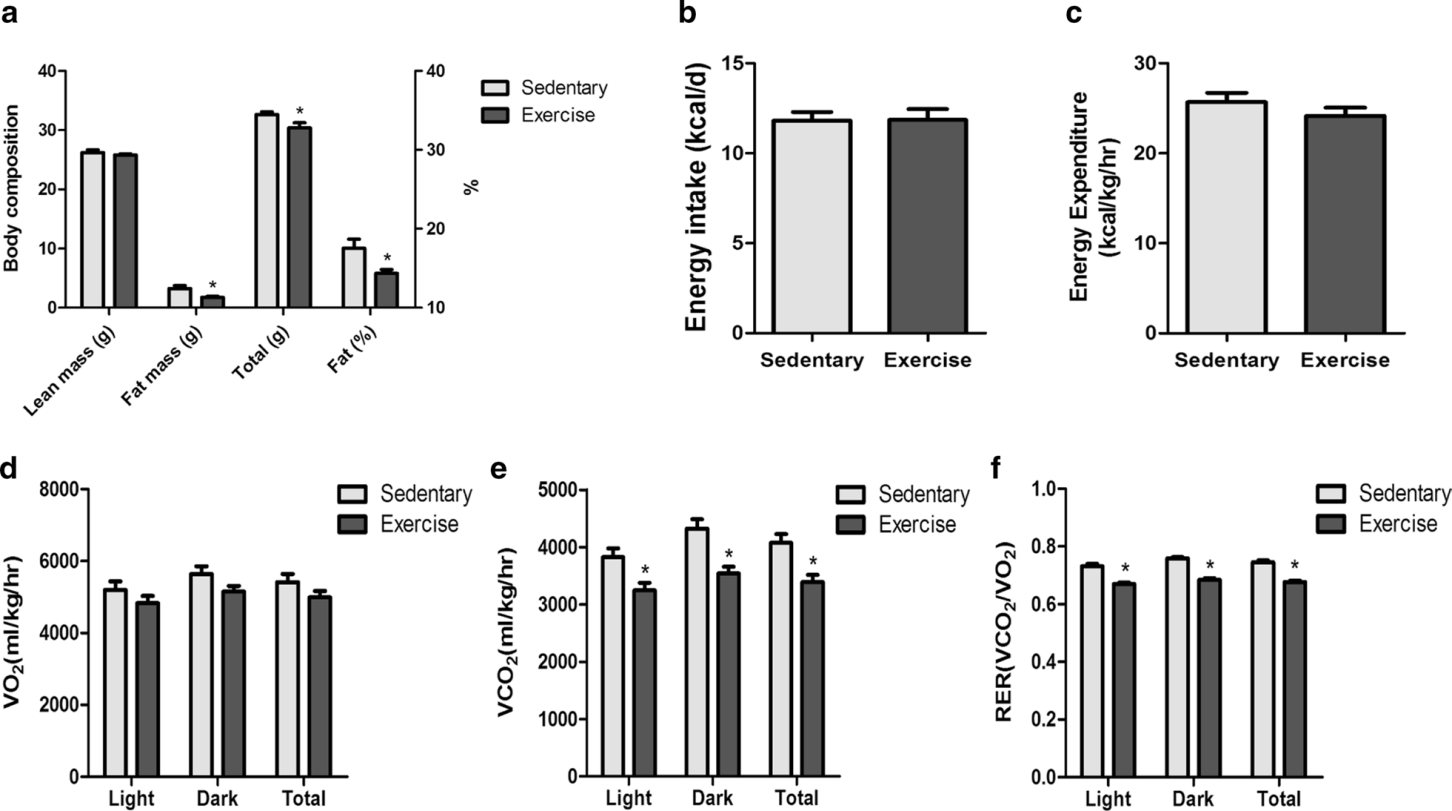
Metabolic parameters of the exercise group compared to the sedentary group. **a** Body composition, **b** energy intake, **c** energy expenditure, **d** average VO_2_, **e** average VCO_2_, and **f** average 24 h RER in the exercise group compared to the sedentary group. n = 16 per group, *p < 0.05

A long-term aerobic exercise training mouse model with moderate-intensity was used to analysis the effect of exercise on weight, body compose, and metabolic parameters. Compared with the mice in the sedentary group, those in the exercise group showed lower body weight (p < 0.05) and fat percentages (p < 0.05) after 12-week exercise training (Fig. 2a). In addition, the VCO_2_ (p < 0.01) and RER (p < 0.001) decreased in the exercise group compared to that in the sedentary group (Fig. 2e and f). But there is no statistical difference in energy intake, energy expenditure, and VO_2_ between the exercise group and the sedentary group (Fig. 2b–d).

### Alteration of hippocampal and hypothalamic RNA-sequencing in mice with 12-week exercise training

**Fig. 3:**
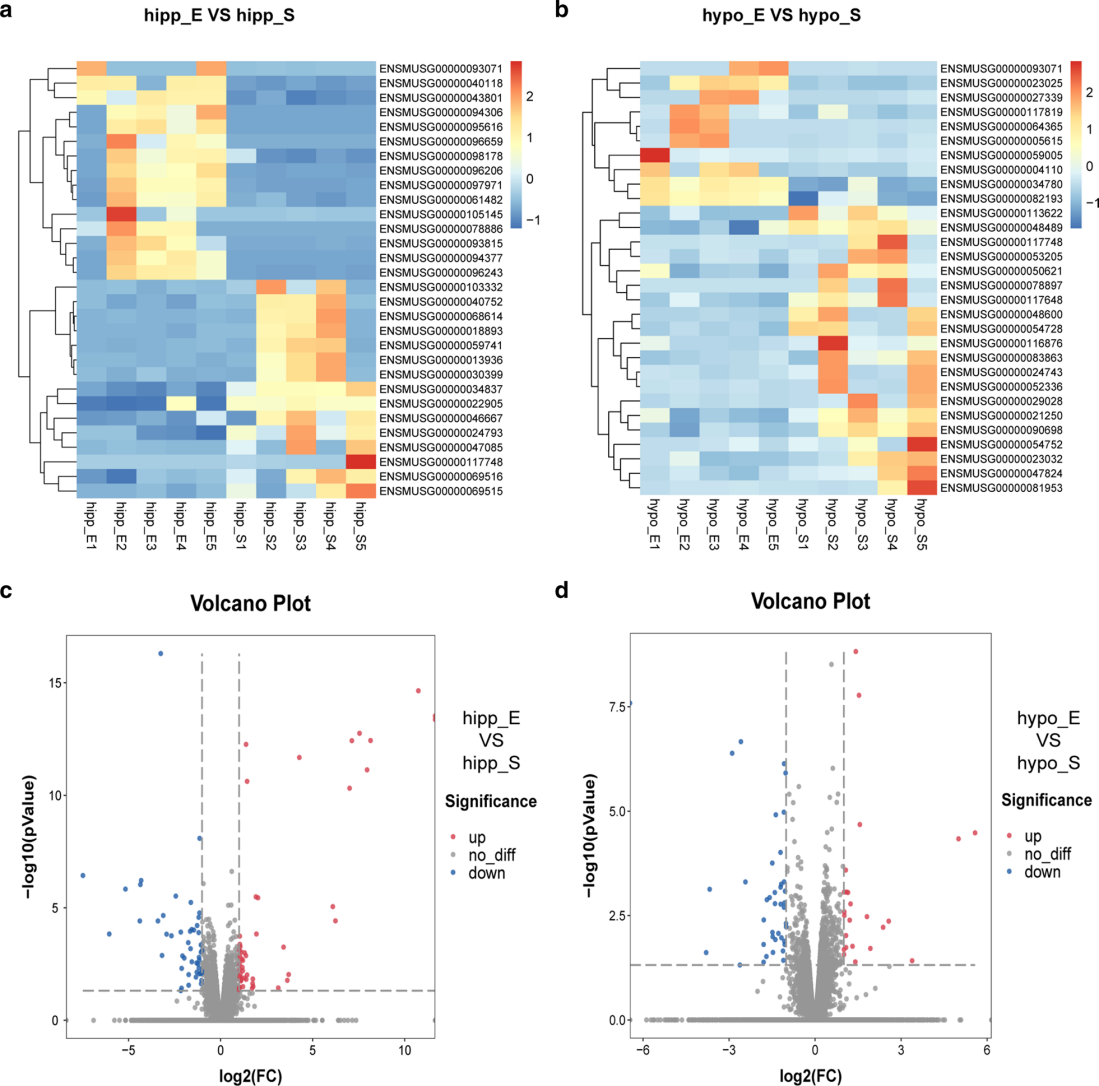
Visualization analysis of differentially expressed genes in hippocampus and hypothalamus. **a**, **b** Heatmap based on RNA-sequencing data of the exercise group compared to the sedentary group in hippocampus and hypothalamus, respectively. **c**, **d** Volcano map based on RNA-sequencing data of the exercise group compared to the sedentary group in hippocampus (up-regulated: 53, down-regulated: 49) and hypothalamus (up-regulated: 24, down-regulated: 40), respectively. Red, blue, and white colors respectively represent the relatively high, low, and equal expression in heatmaps and volcano maps. n = 5 per group, *p* < 0.05

RNA-sequencing determined the transcriptome and expression profiles in the hippocampus and hypothalamus of 5 mice with 12-week exercise training (exercise group) and 5 control mice (sedentary group). Heatmaps and volcano maps showed significant differentially expressed genes in the hippocampus and hypothalamus between the two groups (Fig. 3). As shown in Fig. 3c, 102 differentially expressed genes were observed in the hippocampus: 53 and 49 genes were up-regulated and down-regulated, respectively, in the exercise group compared to that in the sedentary group. However, 64 differentially expressed genes were observed in the hypothalamus: 24 and 40 genes were up-regulated and down-regulated, respectively, in the exercise group compared to that in the sedentary group (Fig. 3d).

**Fig. 4:**
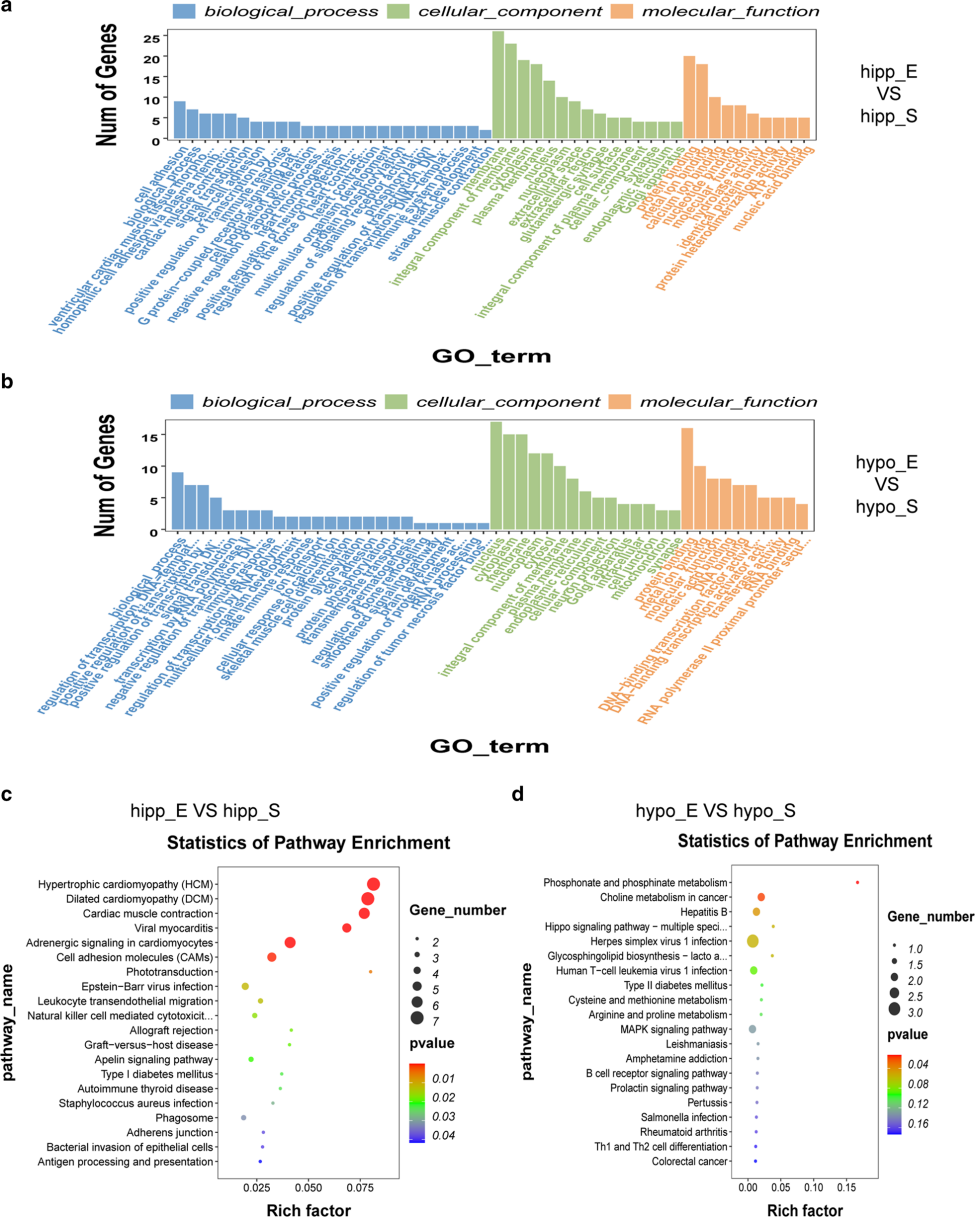
Visualization analysis of GO and KEGG of the exercise group compared to the sedentary group in hippocampus and hypothalamus. **a**, **b** GO analysis classified regulators into BP, CC, and MF groups. **c**, **d** KEGG pathway enrichment. n = 5 per group, *p* < 0.05

Genes with significant changes (|log_2_-fold-change| > 1 and normalized *p* < 0.05) were identified in the exercise and sedentary group, and their functions were annotated using GO and KEGG pathway analyses [16]. The results reported that differentially expressed genes in the hippocampus majorly enriched in biological process (BP), including “cell adhesion,” “biological process” and “ventricular cardiac muscle tissue morphogenesis”; cellular component (CC), including “membrane,” “integral component of membrane” and “cytoplasm”; and molecular function (MF), including “protein binding,” “metal ion binding,” and “calcium ion binding” (Fig. 4a). In addition, differentially expressed genes in the hypothalamus majorly enriched in BP, including “biological process,” “regulation of transcription, DNA-templated,” and “positive regulation of transcription by RNA polymerase II”; CC, including “nucleus,” “cytoplasm,” and “membrane”; and MF, including “protein binding,” “metal ion binding,” and “molecular function” (Fig. 4b). Furthermore, genes involved in the KEGG pathways of the hippocampus enriched “hypertrophic cardiomyopathy signaling pathway,” “dilated cardiomyopathy signaling pathway,” “cardiac muscle contraction signaling pathway,” “adrenergic signaling in cardiomyocytes signaling pathway,” “viral myocarditis signaling pathway,” and “cell adhesion molecules signaling pathway” (Fig. 4c). Similarly, genes involved in the KEGG pathway of the hypothalamus enriched “phosphonate and phosphinate metabolism signaling pathway,” “choline metabolism in cancer signaling pathway,” and “hepatitis B signaling pathway” (Fig. 4d).

### Increased m^6^A level and down-regulated FTO expression in the hippocampus and hypothalamus of mice with 12-week exercise training

**Fig. 5:**
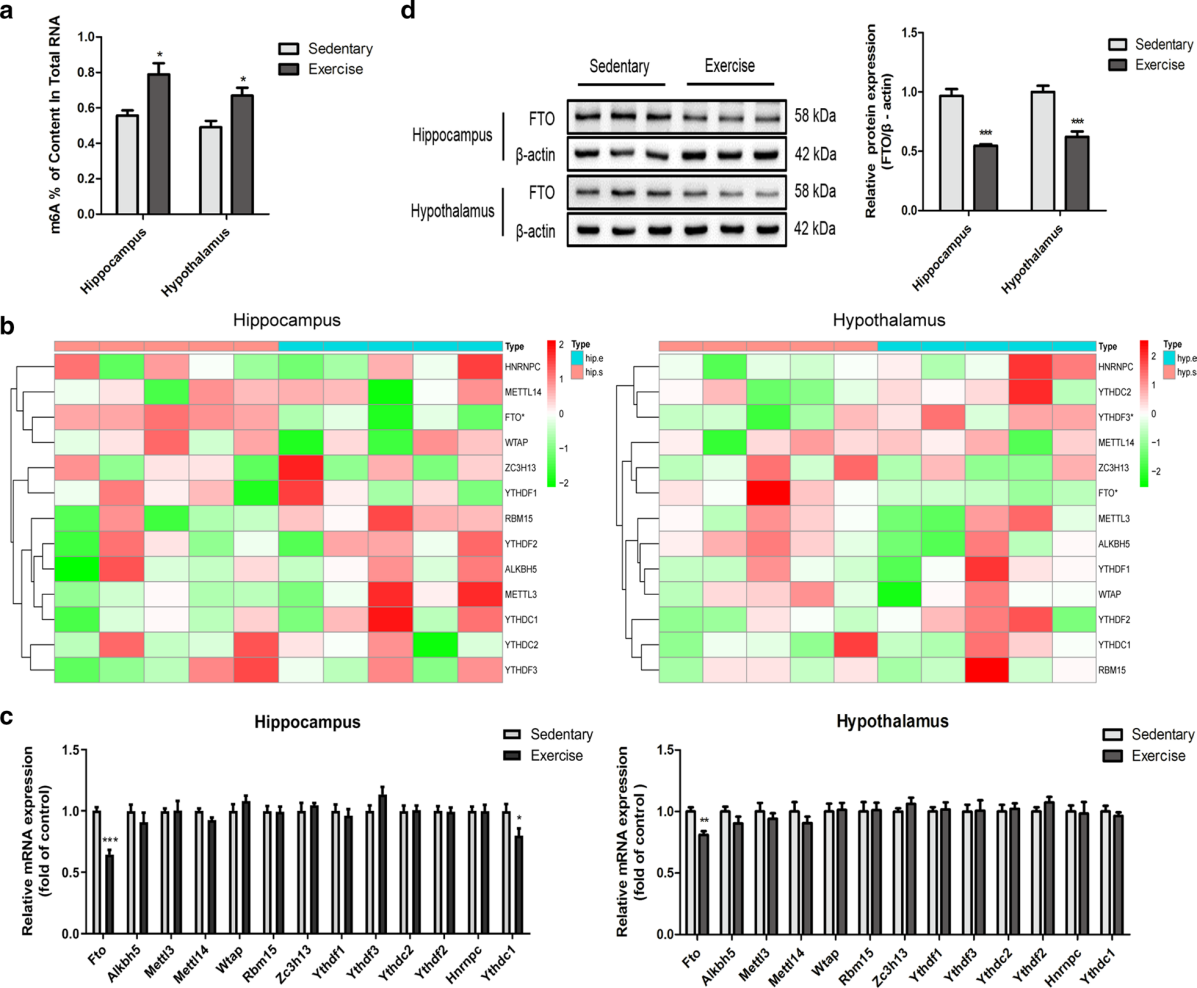
Exercise increased the level of m^6^A and down-regulated FTO expression in hippocampus and hypothalamus. **a** The level of m^6^A was detected using the ELISA method. n = 6 per group, *p < 0.05. **b** The heatmaps showed 13 m^6^A RNA methylation regulators in hippocampus and hypothalamus of the exercise group compared to the sedentary group. hip.E and hyp.E were marked with blue, hip.S and hyp.S were marked with red, position of white spots on the way represented the median value of expression, n = 5 per group, *p* < 0.05. **c** qPCR showed 13 m^6^A RNA methylation regulators mRNA expression. The relative levels of these genes were normalized to *β-actin*, and the relative mRNA levels in the sedentary group were normalized as “1”. n = 6 per group, ***p < 0.01, ***p < 0.001. **d** Western blot analysis of FTO protein levels. The relative levels of FTO were normalized to β-actin, and the relative protein levels in the sedentary group were normalized as “1”. n = 6 per group, ***p < 0.001

Exercise as a positive lifestyle intervention may regulate the downstream genes and various biological processes by changing the RNA methylation. Considering this, the level of m^6^A was detected using ELISA. A high level of m^6^A was observed in hippocampus (p < 0.05) and hypothalamus (p < 0.05) of mice in the exercise group (Fig. 5a), indicating that exercise increased the level of m^6^A in the hippocampus and hypothalamus.

To determine the reason of these changes, the transcriptome and expression profiles of 13 m^6^A RNA methylation regulators in hippocampus and hypothalamus of mice were compared between both the groups. Rank sum test was used to analyze the statistically significant differences, and the results were shown using heatmaps (Fig. 5b). Comparative analyses and qPCR (Fig. 5b and c) showed low *Fto* (*p* < 0.001) and *Ythdc1* (*p* < 0.05) mRNA expressions in the hippocampus and *Fto* (*p* < 0.01) mRNA expression in the hypothalamus of mice in the exercise group, respectively. Further, the level of FTO were detected using western blot, revealing significant downregulation of FTO expressions in the hippocampus (p < 0.001) and hypothalamus (p < 0.001) of mice in the exercise group (Fig. 5d).

### Potential roles of exercise-downregulated FTO in the brain

**Fig. 6:**
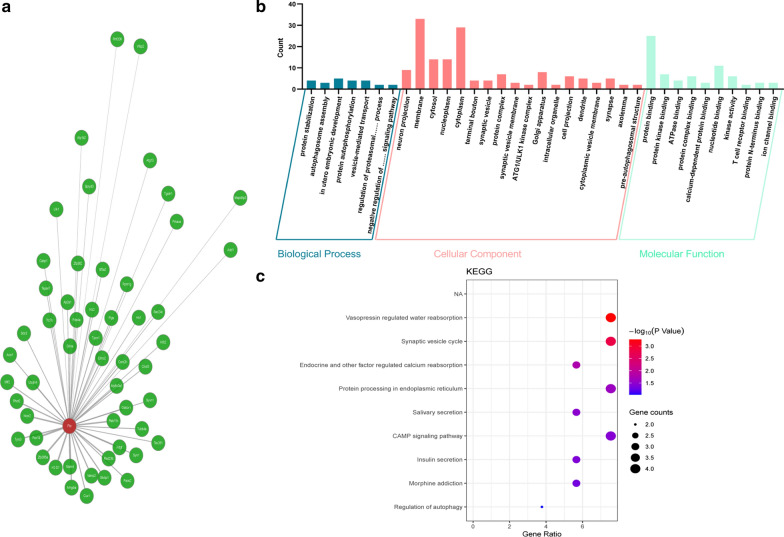
Visualization analysis of *Fto* interaction network, GO and KEGG in the mouse brain. **a** The red ellipse represents *Fto* gene, and the green ellipses represent the *Fto* gene co-expression network. **b** GO analysis classified the co-expression network into BF, CC, and MF terms. **c** KEGG pathway enrichment

Several studies have suggested the importance of FTO in modulating brain functions. However, the genes interacting with FTO were unknown. Hence, the *Fto* gene co-expression network of the mouse brain was analyzed using the coexpedia database to explore the potential role of FTO in the brain. The results showed 54 co-expression genes of *Fto* (Fig. 6a and Table 2). The *Fto*/co-expression genes majorly enriched BP, including “in utero embryonic development,” “protein stabilization,” and “protein autophosphorylation”; CC, including “membrane,” “cytoplasm,” and “nucleoplasm”; and MF, including “protein binding,” “nucleotide binding,” and “protein kinase binding” (Fig. 6b). Furthermore, *Fto*/co-expression genes involved in the KEGG pathway of the hypothalamus enriched “vasopressin regulated water reabsorption signaling pathway,” “synaptic vesicle cycle signaling pathway,” “protein processing in endoplasmic reticulum signaling pathway,” and “CAMP signaling pathway” (Fig. 6c). Therefore, FTO plays diverse physiological and pathological functions in the brain tissues. Further, exercise may play a role in brain functions and biological processes by regulating the FTO expression.

**Table 2:** *Fto* co-expression genes in the mouse brain

Gene symbol	Description	Entrez ID
*Mark4*	MAP/microtubule affinity-regulating kinase 4	232944
*H2-D1*	Histocompatibility 2, D region locus 1	14964
*Zfp385a*	Zin finger protein 385A	29813
*Pex19*	Peroxisomal biogenesis factor 19	19298
*Arhgdia*	Rho GDP dissociation inhibitor (GDI) alpha	192662
*Vamp2*	Vesicle-associated membrane protein 2	22318
*Ncor2*	Nuclear receptor co-repressor 2	20602
*Rab11b*	RAB11B, member RAS oncogene family	19326
*Rad23b*	RAD23 homolog B, nucleotide excision repair protein	19359
*Ubqln4*	Ubiquilin 4	94232
*Stxbp1*	Syntaxin binding protein 1	20910
*Cux1*	Cut-like homeobox1	13047
*Tyro3*	TYRO3 protein tyrosine kinase 3	22174
*Hdgf*	Heparin binding growth factor	15191
*Rhot2*	Ras homolog family member T2	214952
*Atp6v0a1*	ATPase, H+ transporting, lysosomal V0 subunit A1	11975
*Gabbr1*	Gamma-aminobutyric acid (GABA) B receptor, 1	54393
*Grina*	Glutamate receptor, ionotropic, N-methyl D-aspartate-associated protein 1	66168
*Elmo2*	Engulfment and cell motility 2	140579
*Tpcn1*	Two pore channel 1	252972
*Syn1*	Synapsin I	20964
*Mlf2*	Myeloid leukemia factor 2	30853
*Coro2b*	Coronin, actin binding protein, 2B	235431
*Pacs2*	Phosphofurin acidic cluster sorting protein 2	217893
*Tubb4a*	Tubulin, beta 4A class IVA	22153
*Pde4a*	Phosphodiesterase 4A, cAMP specific	18577
*Acin1*	Apoptotic chromatin condensation inducer 1	56215
*Ttc7b*	Tetratricopeptide repeat domain 7B	104718
*Sdc3*	Syndecan 3	20970
*Syvn1*	Synovial apoptosis inhibitor 1, synoviolin	74126
*Klc2*	Kinesin light chain 2	16594
*Ap2a1*	Adaptor-related protein complex 2, alpha 1 subunit	11771
*Pigs*	Phosphatidylinositol glycan anchor biosynthesis, class S	276846
*Cnot3*	CCR4-NOT transcription complex, subunit 3	232791
*Tex261*	Testis expressed gene 261	21766
*Tspan7*	Tetraspanin 7	21912
*Hk1*	Hexokinase 1	15275
*Kifc2*	Kinesin family member C2	16581
*Zfp362*	Zinc finger protein 362	230761
*Ppm1g*	Protein phosphatase 1G (formerly 2C), magnesium-dependent, gamma isoform	14208
*Sf3a2*	Splicing factor 3a, subunit 2	20222
*Sec24c*	Sec24 related gene family, member C (S. cerevisiae)	218811
*Cabp1*	Calcium binding protein 1	29867
*Ulk1*	Unc-51 like kinase 1	22241
*Spryd3*	SPRY domain containing 3	223918
*Prkaca*	Protein kinase, cAMP dependent, catalytic, alpha	18747
*Tgoln1*	Trans-Golgi network protein	22134
*Tpcn1*	Two pore channel 1	252972
*Add1*	Adducin 1 (alpha)	11518
*Atg13*	Autophagy related 13	51897
*Atp1b2*	ATPase, Na+/K+ transporting, beta 2 polypeptide	11932
*Mapk8ip3*	Mitogen-activated protein kinase 8 interacting protein 3	30957
*Wbp2*	WW domain binding protein 2	22378
*Rnf208*	Ring finger protein 208	68846

## Discussion

Physical exercise has substantial beneficial effects not only on physical health but also on brain function. Most studies suggested the importance of exercise on the brain, particularly the hippocampus and hypothalamus. For example, long-term exercise can prevent cognitive dysfunction induced by obesity [17] or aging [18] and improve spatial learning and memory ability. Endurance exercise can alter the gene expression status of the hippocampus, thereby affecting human cognitive function [19]. In addition, exercise ameliorates the hypothalamic leptin resistance [20] and insulin resistance [21] to affect the energy balance. However, the molecular mechanisms through which exercise affects brain function are unclear. The development of high-throughput sequencing provides a beneficial tool to study the role of exercise in regulating the biological processes in the brain by altering the gene expressions.

The molecular mechanisms of exercise that regulates brain function were investigated. We used an exercise mouse model to observe the effects of exercise on gene expression in the hippocampus and hypothalamus. Using high-throughput sequencing technology, differential genes were found to be involved in many important cellular functions and signaling pathways. For example, some enriched functions of the differentially expressed genes in the hippocampus were associated with the synaptic transmission process (GO: 0099025, GO: 0099029, GO: 0099576, GO: 0060080, GO: 0099151, and GO: 0051932), indicating that exercise may regulate synaptic activity. In addition, some enriched functions of the differentially expressed genes in the hypothalamus were associated with the neural function (GO: 0032809 and GO: 0043005), neurogenesis (GO: 0021626 and GO: 0014037), and glucagon secretion regulation (GO: 0070029), suggesting that exercise promotes hypothalamic health and its function. Regarding the KEGG pathway, the “cell adhesion molecules signaling pathway” plays a crucial role in the hippocampal neuronal survival, differentiation, axonal growth, and synaptic development [22, 23].

Recently, the significance of epigenetic regulation in various biological functions and disease pathogenesis has increased. As an epigenetic marker, the reversible m^6^A is the most prevalent post-transcriptional regulation of mammalian gene expression. m^6^A is abundant in the nervous system, and the cellular dynamics of m^6^A are associated with neural function, neurogenesis, and neuronal survival [24–26]. The dysregulation of m^6^A is related to many biological processes, including neurodevelopment and neurodegenerative diseases. Reportedly, the upregulation of m^6^A occurs with brain maturation [27], behavioral experience [28], and memory formation [29]. In this study, a high level of m^6^A was observed in the hippocampus and hypothalamus of mice in the exercise group (Fig. 5a). Since the dynamic equilibrium of m^6^A is governed by m^6^A-related components, such as methylesterases, demethylases, and reading proteins, the expression of 13 m^6^A RNA methylation regulator genes, including *METTL3*, *METTL14*, *WTAP*, *RBM15*, *ZC3H13*, *FTO*, *ALKBH5*, *YTHDF1*, *YTHDF3*, *YTHDC2*, *YTHDF2*, *YTHDC1*, and *HNRNPC* were analyzed in the hippocampus and hypothalamus of mice in the exercise group. The result showed that only *Fto* was down-regulated in the hippocampus and hypothalamus of the mice in exercise group (Fig. 5b and c). In addtion, western blot experiment was performed, confirming the finding (Fig. 5d).

FTO as an m^6^A demethylase is a crucial component of m^6^A modification [30, 31]. Several studies suggested that *FTO* knockdown with siRNA increased the amount of m(^6^)A in mRNA, and *FTO* overexpression decreased the amount of m(^6^)A in human cells [12]. The above evidence proves that FTO expression may contribute to m^6^A levels. Hence, presumably, elevated levels of m^6^A in the hippocampus and hypothalamus after exercise are due to the downregulation of FTO. Although polymorphisms within the intron 1 of the *FTO* gene were first reported to be associated with obesity [10, 32, 33], the physiological role of the *FTO* gene remains unclear. FTO is widely found in central and peripheral tissues of mammals [34]. In peripheral tissues, FTO is related to energy metabolism [35, 36] and cancer progression [37–39]. In central tissues, FTO is highly expressed in the brain and essential for development of the central nervous system in humans [40, 41]. Numerous preclinical evidence reported that altered FTO expression is partially responsible for energy balance, epilepsy, neurodevelopment, and neurodegenerative diseases. In animal studies, FTO can activate the phosphorylation of Tau, which is one of the markers of Alzheimer's disease (AD) [42]. In human studies, the genetic variation in the introns of the *FTO* gene possibly contributes to the risk of AD [43, 44]. However, specific mechanism of the *FTO* gene variants that contribute to the risks of AD is still unclear and requires further research. Moreover, the FTO inhibitor can regulate the neuronal excitability with anticonvulsant activity [45],and is responsible for glioblastoma progression [46]. Axonal FTO is reportedly involved in neuronal development by regulating the m^6^A modification of axonal mRNA [47]. Decreasing FTO in the dorsal hippocampus aids in memory formation [29]. However, the loss of FTO leads to impairment of neuronal differentiation and a processing defect of brain-derived neurotrophic factor (BDNF) within the hippocampus, which increasing anxiety and impairing the working memory [48]. In addition, the complete or neural-specific *Fto* gene deletion results in postnatal growth retardation of mice [34]. The m^6^A RNA demethylase FTO alleviates the deficits in dopaminergic neurotransmission in response to arsenite exposure [49]. FTO is related to appetite and food intake in the hypothalamus [50]. Further research found that mice with low expression of FTO remain sensitive to the anorexigenic effects of leptin [51]. All these studies strongly suggest that FTO plays vital roles in the physiological and pathological functions of the brain.

Although most studies have focused on the impact of FTO overexpression or knockdown in the brain, the genes that related to FTO are still important as they perform many subsequent molecular functions and biological processes. It has been reported that FTO as a transcriptional coactivator promotes gene transcription, ultimately affecting adipose tissue development [36]. However, the mechanism of FTO interaction with downstream genes to further regulate nerve function remain largely unknown.

FTO could be regulated not only by nutrition but also by exercise. Previous studies found that physical activity might weaken the effect of the *FTO* variant on BMI [52–56]. In addition, gender also influences the *FTO* genotype on exercise for weight loss. It is observed that males carrying the *FTO* risk allele lose more weight after a 12-week regular exercise [57]. An acute decreased skeletal muscle *FTO* mRNA expression was observed after high-intensity exercise by Danaher et al. [14]. Most researchers focus on the reducing obesity risk caused by *FTO* gene polymorphisms under exercise, while there are few on its function. In the present study, we indicated that exercise attenuates FTO expression. FTO, a demethylase, plays an important role in energy metabolism. The abnormal FTO expression modifies the level of m^6^A of target genes and is involved in many physiological and pathological processes. Overall, the study reported for the first time that long-term exercise can down-regulated the FTO expression in the hippocampus and hypothalamus, indicating that FTO may be a promising key player between exercise and the brain.

However, it is unclear whether exercise-induced FTO downregulation can regulate downstream target genes and the biological processes. Hence, the *Fto*/co-expression genes were downloaded from the database for GO enrichment and KEGG signal pathway analyses. Based on the results of bioinformation analyses, the significant enrichment pathway primarily correlated with vasopressin-regulated signaling pathway, water reabsorption signaling pathway, synaptic vesicle cycle signaling pathway, endocrine signaling pathway, calcium reabsorption signaling pathway, protein processing in endoplasmic reticulum signaling pathway, salivary secretion signaling pathway, cAMP signaling, insulin secretion signaling pathway, and morphine addiction signaling pathway (Fig. 6c). The result suggests that FTO and its co-expression genes are involved in many important biological processes in the brain. In addtion, the known and unknown proteins co-expressed with FTO may be regulated by FTO-m^6^A to alter their expression and function. Thus, exercise may regulate the expression and function of the related genes via FTO-dependent demethylation of mRNA m^6^A.

We found that the *Vamp2* gene expression in mouse brain is involved in two KEGG signaling pathways, include insulin secretion and synaptic vesicle cycle. Vesicle-associated membrane protein 2 (VAMP2) has been implicated in the insulin-regulated trafficking of GLUT4 in insulin-sensitive cells. VAMP2 inhibited insulin-stimulated GLUT4 translocation and decreased insulin sensitivity [58, 59]. Insulin-sensitive tissue or cells include liver, skeletal muscle, adipocytes, and hypothalamus. In addition, VAMP2 may have important roles in synaptic trafficking in the hippocampus [60]. The epileptogenesis is dramatically attenuated in hippocampus of *Vamp*^+/−^ mice [61]. Hence, presumably, long-term exercise may regulate the expression and function of VAMP2 in hypothalamus and hippocampus via FTO-dependent demethylation of mRNA m^6^A, but further research is needed to confirm that VAMP2 is a target of FTO. Hence, FTO could be a valuable therapeutic target for brain diseases in the future.

## Conclusion

The gene changes after exercise training were confirmed using RNA-sequencing analysis. Long-term exercise training showed increased level of m^6^A and down-regulated FTO expressions in the hippocampus and hypothalamus. Lifestyle intervention such as exercise might be an effective intervention for epigenetic modification. In addition, reviewed of studies on the role and co-expression genes of *Fto* in mice brain revealed that the relationship between FTO and downstream genes is not completely reported, requiring additional research to elucidate their roles in the brain in response to exercise. Nevertheless, further research is warranted to understand the signaling pathways of FTO involved in and their impacts on brain health.

## Data Availability

The RNA-sequencing datasets used and/or analyzed during the current study are available from the corresponding author on reasonable request. The data used to bioinformatic analysis of *Fto* gene co-expression network can be accessed at coexpedia database: https://www.coexpedia.org/mm_single.php?gene=FTO.

## Abbreviations

m^6^A: N6-methyladenosine
FTO: Fat mass and obesity-associated gene
VO_2_: Oxygen consumption
VCO_2_: Carbon dioxide production
RER: Respiratory exchange ratio
GO: Gene ontology
KEGG: Kyoto encyclopedia of genes and genomes
mRNA: Messenger RNA
METTL3: Methyltransferase-like 3
METTL14: Methyltransferase-like 14
WTAP: Wilms' tumor 1 associated protein
RBM15: RNA binding motif protein 15
ZC3H13: Zinc finger CCCH domain-containing protein 13
ALKBH5: Alkylation repair homolog protein 5
YTHDF1: YTH domain-containing family protein 1
YTHDF3: YTH domain-containing family protein 3
YTHDC2: YTH domain-containing protein1
YTHDF2: YTH domain-containing family protein 2
YTHDC1: YTH domain-containing protein1
HNRNPC: Heterogeneous nuclear ribonucleoprotein C
AD: Alzheimer’s disease
BDNF: Brain-derived neurotrophic factor
